# Study of the Complexation Behaviour of Fexofenadine with β-Cyclodextrin

**DOI:** 10.4103/0250-474X.70477

**Published:** 2010

**Authors:** Nidhi P. Sapkal, Vaishali A. Kilor, Bharti D. Shewale, K. P. Bhusari, A. S. Daud

**Affiliations:** Gurunanak College of Pharmacy, Near Dixit Nagar, Nari, Nagpur-440 026, India; 1 Sharad Pawar College of Pharmacy, Wanadongri, Nagpur-441 110, India; 2 Zim Laboratories Ltd. Kalmeshwar, Nagpur-441 501, India; 3 H. R. Patel women’s College of Pharmacy, Shirpur-425 405, India

**Keywords:** β-cyclodextrin, kneading, co-precipitation, fexofenadine, inclusion complexes

## Abstract

Fexofenadine is a selective histamine H_1_ receptor antagonist, used for relief of the symptoms of allergy. However its aqueous solubility is very poor. Solid inclusion complexes of fexofenadine and β-cyclodextrin were prepared at the molar ratios of 1:1 and 1:2 by kneading, and coprecipitation methods to improve its solubility. Characterization of the complexes was performed using infrared spectroscopy, X-ray diffractometry, and *in vitro* dissolution studies. Fexofenadine was found to exhibit interaction with β-cyclodextrin both in solid and liquid state. Phase solubility studies indicated that fexofenadine forms a stable complex with β-cyclodextrin. Both IR spectroscopy and X-ray diffractometry studies indicated interaction of fexofenadine with β-cyclodextrin. Kneading method at 1:1 and co-precipitation method at 1:1 and 1:2 molar ratios showed significant interaction. *In vitro* dissolution studies confirmed the same results.

Fexofenadine hydrochloride (FFN) is the synthetic hydrochloride salt of fexofenadine, the carboxylic acid metabolite of terfenadine. High plasma concentrations of terfenadine have been associated with rare incidences of cardiac arrhythmias and this was the reason for the withdrawal of drug from clinical use. FFN has been promoted as terfenadine’s replacement. It is an orally active non-sedating H_1_-receptor antagonist and is effective for the relief of symptoms associated with seasonal allergic rhinitis. It is superior to most other antihistamines as it does not cross blood brain barrier and is a safe antihistamine[1]. But FFN is very slightly soluble in water[2], and poor aqueous solubility of a drug is the factor that limits its development into desired formulation. The absorption of such drugs may be incomplete and variable and hence there will be variations in the effectiveness[3].

There have not been many attempts to enhance the solubility of FFN. Fast disintegrating granules[4] and fast dissolving complex[5] of FFN with carbomer has been reported. A mucoadhesive nasal and opthalmic delivery system is proposed in a patent application for the controlled delivery of FFN. This system utilizes the hydroxypropyl-β-cyclodextrin as solubility enhancer but there is no mention of formation of inclusion complexes[6]. An attempt to study the complexation between FFN and β-cyclodextrin (β-CD) has been done recently[7]. Cyclodextrins (CDs) have been found to be very useful in enhancing the solubility of poorly water-soluble drugs owing to the formation of inclusion complex of the drug in its hydrophobic cavity[8–12]. The most common natural CDs are α-CD, β-CD and γ-CD. These differ from each other in number of glucose units. Amongst these β-CD has been found to be cost effective for the development of commercial formulations. Moreover, β-CD is nontoxic for oral use[13].

The present work was designed to improve the aqueous solubility of FFN. The enhanced aqueous solubility of a drug increases the avenues for the development of variety of dosage forms and improves the bioavailability. Before proceeding with the formation of complexes, phase solubility analysis was carried out to determine the feasibility of complex formation between FFN and β-CD. After determining the stability constant value, the inclusion complexes of FFN were prepared using simple methods of inclusion complex formation like kneading and co-precipitation in different molar ratios. Physical mixtures of FFN and β-CD were also prepared for comparison purpose. The so prepared complexes were then characterized using techniques like infrared-spectroscopy, X-ray diffractometry and *in vitro* dissolution studies.

## MATERIALS AND METHODS

Both β-CD and FFN were received from Zim Laboratories Ltd., Nagpur, India, as gift samples. All the reagents and solvents were of analytical grade, and double distilled water was used.

### Phase solubility studies:

Phase solubility studies were carried out according to the method reported by Higuchi and Connors[14]. A series of solutions of β-CD of concentrations ranging from 0.001 mol-0.05 mol in phosphate buffer (pH 6.8) were prepared. Each of this solution (50 ml) was added into a series of 250 ml stoppered conical flasks. A constant amount of pure drug (50 mg) was accurately weighed and suspended into each of these solutions. The solutions were then shaken in constant temperature bath shaker at 25±0.5°. Aliquots (2 ml) were withdrawn at 12 h intervals and filtered with membrane filter (0.2 μ). The filtrates were diluted suitably, if required, with phosphate buffer and analyzed by HPLC (HPLC-UV100, Spectra-Physics Analytical Inc., Manchester, UK) for determining FFN content. Shaking was continued until 3 consecutive estimations were the same (72 h). The solubility experiments were conducted in triplicate. The phase solubility diagram of drug and β-CD was obtained by plotting the concentration of drug against β-CD concentration.

### Formulation of inclusion complexes by physical mixing method:

FFN and β-CD were blended in the mortar for 30 min in the molar ratios of 1:1 and 1:2 and were given the names FPM1 and FPM2 respectively (Table 1). These fine powdered physical mixtures were then stored in the desiccator containing anhydrous silica gel for comparison with the corresponding solid complex powders.

### Formulation of inclusion complexes by kneading method:

Weighed quantity of β-CD was mixed with minimum quantity of distilled water in a mortar so as to obtain a homogeneous paste. Weighed quantity of drug powder was then added slowly. The mixture was then grounded for 1 h. During this process, an appropriate quantity of water was added to the mixture in order to maintain a suitable consistency. The whole procedure was carried out at room temperature. The paste was dried in oven at 45° for 24 h. The dried complex was pulverized, and passed through No. 80 sieve and stored in desiccator at ambient temperature till further studies. The complexes were prepared in both 1:1 and 1:2 molar ratios and were given the names FK1 and FK2 respectively (Table 1).

### Formulation of inclusion complexes by co-precipitation method:

The complexes by co-precipitation method were also prepared in both 1:1 and 1:2 molar ratios and were given the names FCP1 and FCP2 respectively (Table 1). First β-CD was dissolved in distilled water with the help of heat. FFN powder was separately dissolved in minimum quantity of methanol. Drug solution in methanol was then added to aqueous solution of β-CD slowly under stirring at room temperature. After complete addition the mixture was maintained at 70° while being stirred continuously with the help of mechanical stirrer for 1 h. The solution was then allowed to cool gradually and kept for digestion overnight. The co-precipitates were then filtered and dried overnight in a vacuum dessicator. The dried binary mixtures were passed through No. 80 sieve and stored in desiccator until further evaluation.

**TABLE 1:** FORMULATIONS OF BINARY MIXTURES OF FFN WITH B-CD

Formulation code	Drug (g)	β-CD (g)
Physical mixing method		
FPM1	FFN (5.0)	11.35
FPM2	FFN (5.0)	22.70
Kneading method		
FK1	FFN (5.0)	11.35
FK2	FFN (5.0)	22.70
Co-precipitation method		
FCP1	FFN (5.0)	11.35
FCP2	FFN (5.0)	22.70

FPM1 and FPM2 are binary mixtures prepared by physical mixing in 1:1 and 1:2 molar ratios, respectively; FK1 and FK2 are the binary mixtures prepared by kneading method in 1:1 and 1:2 molar ratios, respectively; FCP1 and FCP2 are the binary mixtures prepared by co-precipitation method in 1:1 and 1:2 molar ratios, respectively

### Drug content measurements:

The binary mixtures that were prepared by physical mixing, kneading and co-precipitation of FFN i.e., FPM1, FPM2, FK1, FK2, FCP1 and FCP2 were assayed. Powder of each binary mixture (50 mg) was accurately weighed and transferred to an Erlenmeyer flask containing methanol (50 ml). These flasks were then ultrasonicated for 15 min and filtered. The filtrate was added into volumetric flask (100 ml) and volume was made up with methanol. These solutions were then analyzed for drug content by measuring the area under curve by running the sample through HPLC. The percent drug content was calculated and compared with percent theoretical drug content.

### IR spectroscopy:

The interaction between FFN and β-CD were studied by FTIR spectroscopy. The FTIR spectra required were taken from dried samples of pure FFN, and of its binary mixtures, using KBr pellet method and compared (FTIR-8101, Shimadzu, Kyoto, Japan). The scanning range was 400-4000 cm^−1^.

### X-ray diffractometry:

The X-ray diffractograms of the prepared binary mixtures and of pure FFN were carried out using X-ray diffraction instrument (X’pert, XRD System PW1710, Philips, Holland). The samples were irradiated with monochromatized Ni-filtered Cu radiation, and analyzed between 2θ angles of 4° and 54°. The voltage and current per step used were 35 kv and 20 mA, respectively. The scanning speed was 2θ/min and the chart speed was 1 cm/min.

### Dissolution rate studies:

Pure FFN and its binary mixtures were subjected to *in vitro* dissolution studies. These studies were carried out in USP six station dissolution rate test apparatus I (Veego Scientific, DA-6D, Mumbai, India) using 900 ml of phosphate buffer (pH 6.8) as dissolution medium. The powders were tied in a muslin cloth and placed in the basket of stirrer. A speed of 100 rpm and a temperature of 37±0.5° were used for each study. About 10 ml aliquot was withdrawn at different time intervals, filtered using a 0.45-μm nylon disc filter, and replaced with 10 ml of fresh dissolution medium. The filtered samples were suitably diluted if necessary and analyzed for the FFN content. The dissolution experiments were conducted in triplicate.

## RESULTS AND DISCUSSION

**Fig. 1:**
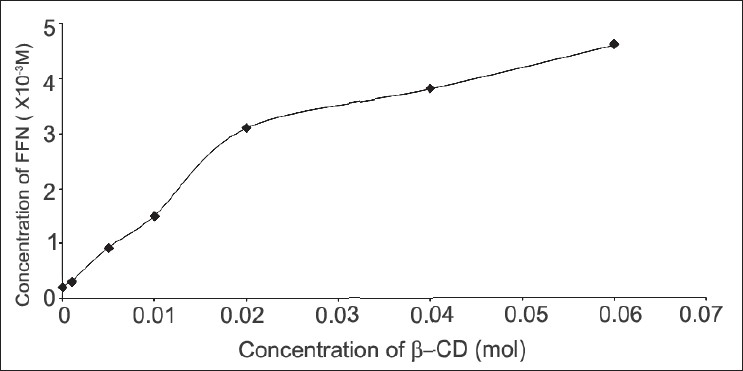
Phase solubility plot for FFN in β-CD Solubility of FFN at different β-CD concentrations (-■-) in pH 6.8 phosphate buffer.

The changes in the solubility of FFN as a function of β-CD concentration are shown in fig. 1. The curve is linear initially and then deviates from linearity in the negative direction. The Ks i.e. stability constant value was calculated according to the equation of Higuchi and Connors[14] from the straight-line portion of the solubility diagram using the Eqn; Ks= slope/ S_o_(1-slope), where S_o_ is the intrinsic solubility of FFN in the absence of β-CD. The value of stability constant was determined to be 2124 M^-1^. When assayed by HPLC, FFN content in all the solid dispersions was found to be sufficiently stable during the processing as depicted by the acceptable assay values (range: 98.67%-99.93%).

**Fig. 2:**
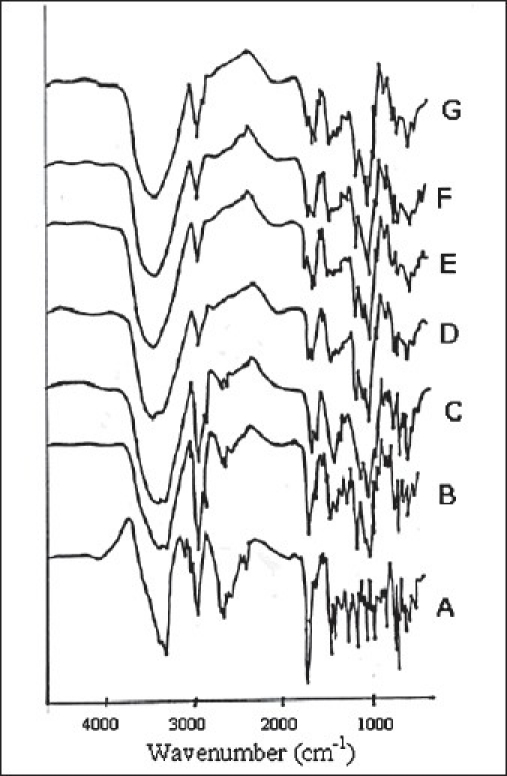
IR spectra of FFN and its binary mixtures. IR spectra of FFN and its binary mixtures; FFN (A), FPM1 (B), FPM2 (C), FK1 (D), FK2 (E), FCP1 (F) and FCP2 (G). FFN is the pure drug; FPM1 and FPM2 are binary mixtures prepared by physical mixing in 1:1 and 1:2 molar ratios; FK1 and FK2 are the binary mixtures prepared by kneading method in 1:1 and 1:2 molar ratios; FCP1 and FCP2 are the binary mixtures prepared by co-precipitation method in 1:1 and 1:2 molar ratios.

The FTIR spectra of pure FFN and of its binary mixtures are given in the fig. 2. Pure FFN is showing peaks at 3298 cm^−1^ (hydroxyl groups), 2937 cm^−1^ (Acid O-H), 1707 cm^−1^ (Carboxylic group), 1655 cm^−1^ (C=C group), 1448 cm^−1^ (butyl chain), 1279 cm^−1^, and 702 cm^−1^ (aromatic rings). Retention of most of the characteristic peaks of FFN is observed during the comparative analysis of IR spectra of FPM1 and FPM2 with FFN. The peak at 3298 cm^−1^ is visible in FPM1 and FPM2 but it is modified greatly in FK1, FK2, FCP1 and FCP2. Peaks at 2937 cm^−1^ and at 1707 cm^−1^ are retained in FPM1 and FPM2 While in FK1, FK2, FCP1 and FCP2 this band became very weak and is shifted to 1718 cm ^-1^. Peak at 1448 cm^−1^ is also found to be retained in all the binary mixtures. The peaks denoting the presence of aromatic groups were modified greatly in both FK2 and FCP2.

**Fig. 3:**
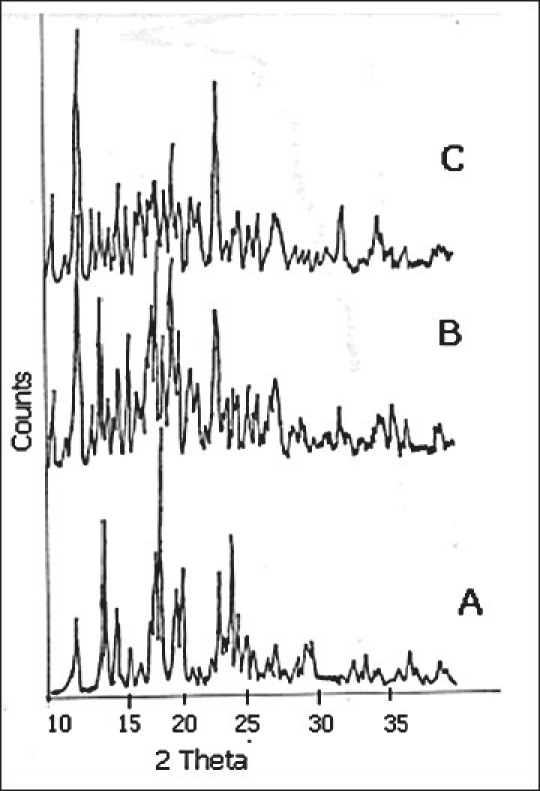
X-ray diffraction patterns of Pure FFN and its physical mixtures X-ray diffraction patterns of FFN and its physical mixtures; FFN (A), FPM1 (B), FPM2 (C). FFN is the pure drug; FPM1 and FPM2 are binary mixtures prepared by physical mixing in 1:1 and 1:2 molar ratios, respectively.

**Fig. 4:**
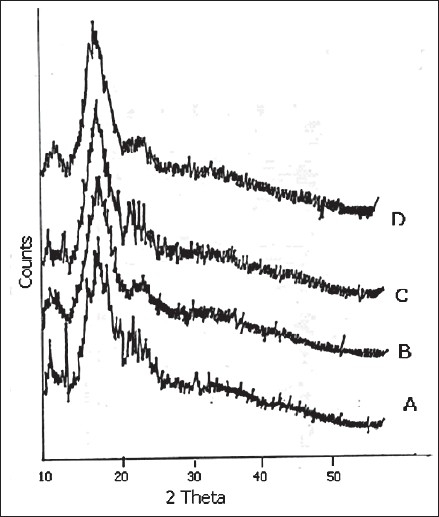
X-ray diffraction patterns of binary mixtures prepared by kneading and co-precipitation. X-ray diffraction patterns of kneaded and co-precipitated binary mixtures; FK1 (A), FK2 (B), FCP1 (C), FCP2 (D). FK1 and FK2 are the binary mixtures prepared by kneading method in 1:1 and 1:2 molar ratios respectively; FCP1 and FCP2 are the binary mixtures prepared by co-precipitation method in 1:1 and 1:2 molar ratios, respectively.

The X-ray diffraction patterns of pure FFN, physical mixtures, kneaded and co-precipitated powders of the FFN and β-CD are represented in figs. 3 and 4. Table 2 depicts the number of peaks and maximum peak intensity for pure FFN and its binary mixtures. The diffractogram of pure FFN exhibited a series of intense peaks, which indicate the crystalline nature of the compound. The diffractogram of the FPM1 and FPM2 are showing more number of peaks and relative intensities of these peaks are higher as compared to the relative intensities of individual peaks of pure FFN. The diffractograms of FK1, FK2, FCP1 and FCP2 differ significantly from the diffractogram of FFN. There is a marked decrease in the number of peaks and maximum peak intensity in all these samples. In the diffractograms of FK1, FK2, FCP1 and FCP2 many of the old peaks of FFN and β-CD are absent and some are less intense. The peaks shown by FFN at 18.3 and 14.12 have undergone a substantial reduction in intensity in FK1, FCP1 and FCP2. While in FK2 it has disappeared. Moreover, both the peaks that are shown by FK2 are new.

**TABLE 2:** X-RAY DIFFRACTOMETRY DATA OF FFN AND ITS BINARY MIXTURES

Formulation code	Total no. of peaks	Maximum peak intensity
FFN Pure	55	655.36
FPM1	63	462.25
FPM2	55	645.16
FK1	15	144.00
FK2	2	16.00
FCP1	12	114.49
FCP2	1	75.69

**Fig. 5:**
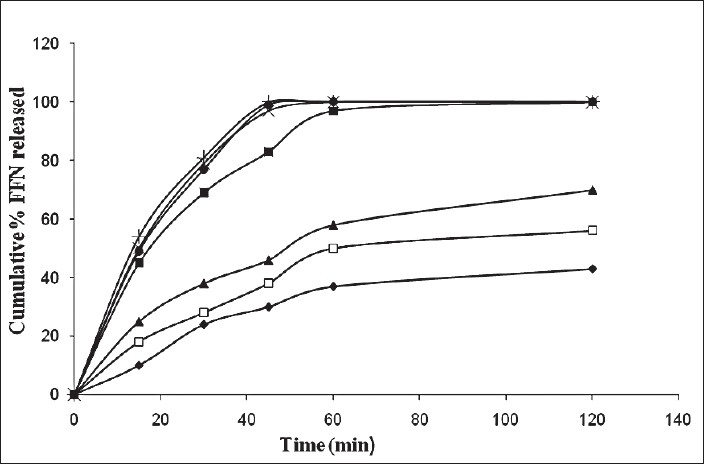
Average cumulative release profile of FFN and its binary mixtures Release profiles of FFN from binary mixtures; FFN (pure drug, -♦-), FPM1 (binary mixtures prepared by physical mixing in 1:1 molar ratios, -□-), FPM2 (binary mixtures prepared by physical mixing in 1:2 molar ratios, -▲-), FK1 (binary mixtures prepared by kneading method in 1:1 molar ratios, -■-), FK2 (binary mixtures prepared by kneading method in 1:2 molar ratios, -×-), FCP1 (binary mixtures prepared by co-precipitation method in 1:1 and 1:2 molar ratios, -●-), FCP2 (binary mixtures prepared by co-precipitation method in 1:2 molar ratios, -+-). Each data point represents mean (n = 3).

Average cumulative release profile of pure FFN and its binary mixtures is shown in fig. 5. The reported values were obtained by calculating the arithmetic mean of three measurements, and standard deviation bars are omitted to avoid overlapping. After 15 min pure drug was only 10% soluble while the physical mixtures showed about 20% dissolution of FFN. FCP2 showed quickest release by dissolving about 55% of the drug in 15 min. In 45 min more than 80% of FFN was released by all the binary mixtures except FPM1 and FPM2. From FPM1 and FPM2 the release was only 38% and 46% respectively. FFN from FK2, FCP1 and FCP2 was completely dissolved in 45 min. The pure drug was released only up to 30% in 45 min.

In phase solubility studies FFN was found to show A_N_ type of curve. This indicates that there is a linear host guest correlation and a complex of constant composition is formed with the increase in concentration of β-CD. At still higher concentrations of β-CD negative curvature indicates towards changes in the nature of complexes owing to different reasons[15]. These results are in agreement with those described by other researchers[7]. Initial linear portion of the curve shows 1:1 stoichiometry between FFN and β-CD which later shifts to 1:2. The value of stability constant indicates the formation of a stable complex in the liquid sate. It has been reported that CD-drug complexes with the values of stability constant in the range of 200 to 5000 M^−1^ show improved bioavailability[14]. The stability constant of 2124 M^−1^ is well with in this range and therefore the inclusion complexes formed between FFN and β-CD can be expected to improve the bioavailability of FFN.

IR spectroscopy studies indicated that both the kneading and co-precipitation method results in the interactions between FFN and β-CD. Presence of all the characteristic peaks of FFN in the IR spectra of FPM1 and FPM2 shows that the physical mixing method does not bring about any chemical interaction between FFN and β-CD. In FK1, FK2, FCP1 and FCP2 the changes in the characteristic band of carboxylic group at 1707 cm^−1^ of FFN indicate that phenyl group attached to carboxylic group is involved in the inclusion complex formation with β-CD. The bands produced by aromatic hydrogens in pure FFN were greatly modified in all the binary mixtures. The significant reduction in the intensity of band at 744 cm^−1^ (aromatic group) shows the inclusion of aromatic groups of FFN into the cavity of β-CD during the formation of binary mixtures. Retention of peak at 1448 cm^−1^ in all the binary mixtures indicates that the butyl chain is not interacting with β-CD in any way. Thus analysis of IR spectra indicated that both the methods i.e. kneading and co-precipitation lead to the interaction of the aromatic rings of FFN with the cavity of β-CD.

In the X-ray diffractometry studies of FFN and its binary mixtures no significant difference in the number of peaks and maximum peak intensity of FFN, FPM1 and FPM2 is found. This depicted that physical mixing of FFN with β-CD did not produce any change in the nature of FFN and its crystalline nature is retained. While FK1, FK2, FCP1 and FCP2 showed dramatic reduction in the number of peaks and in maximum peak intensity. Amongst these batches highest crystallinity is observed in FK1. In FK2 only two and in FCP2 only one peak was detected. But the maximum peak intensity was found to be lowest in the case of FK2 (16.00 counts/sec) amongst all the binary mixtures. Complete amorphization was observed in all of these products. Thus, X-ray diffraction studies of FFN and its binary mixtures suggested that both the kneading and co-precipitation methods in both the molar ratios (i.e. 1:1 and 1:2) were effective in bringing out a noteworthy change in the crystal structure of FFN. But FK2 showed the maximum modification as depicted by number of peaks and maximum peak intensity.

The *in vitro* dissolution studies showed slight improvement in the release of FFN in the case of physical mixtures. This improvement may be due to the ability of β-CD to increase the wettability of drug powder[16]. A significant influence of kneading and co-precipitation method was seen on the dissolution behavior of FFN. The higher dissolution rates observed with FK2, FCP1 and FCP2 may be due to the better interaction of drug and β-CD during the kneading process in 1:2 molar ratio and during the co-precipitation process in both 1:1 and 1:2 molar ratios. This reveals that kneading method results in the formation of inclusion complexes in 1:1 molar ratio while co-precipitation method produces inclusion complexes in both the molar ratios. The results were in agreement with the results of X-ray diffractometry studies. It has been previously demonstrated by ^1^H NMR titration studies that inclusion complexes of FFN with β-CD are possible in both 1:1 and 1:2 molar ratios in aqueous solutions[7]. Existance of these inclusion complexes in solid state can help in the formulation of variety of dosage forms with better dissolution. In the present work it has been observed that the inclusion complexes of FFN with β-CD in the solid state are possible and they result in the enhancement of dissolution.
